# 5,10,15,20-Tetra­kis(1-methyl­pyridinium-4-yl)porphyrin tetra­iodide tetra­hydrate

**DOI:** 10.1107/S1600536811043844

**Published:** 2011-11-02

**Authors:** Leandro M. O. Lourenço, José A. Fernandes, Maria G. P. M. S. Neves, José A. S. Cavaleiro, João P. C. Tomé, Filipe A. Almeida Paz

**Affiliations:** a Department of Chemistry, University of Aveiro, QOPNA, 3810-193 Aveiro, Portugal; b Department of Chemistry, University of Aveiro, CICECO, 3810-193 Aveiro, Portugal

## Abstract

The asymmetric unit of the title compound, C_44_H_38_N_8_^2+^·4I^−^·4H_2_O, comprises two halves of non-equivalent cations of 5,10,15,20-tetra­kis­(1-methyl­pyridinium)porphyrin (with the full mol­ecule of each completed by the application of inversion symmetry), four charge balancing iodide anions and four water mol­ecules of crystallization (two water mol­ecules are fully occupied and four mol­ecules have a site occupancy of 50%). The porphyrin cations are arranged into supramolecular columns parallel to the *b* axis, mediated by π–π [centroid–centroid distance = 3.762 (4) Å] and C—H⋯π supra­molecular inter­actions [C⋯centroid distance = 3.522 (7) Å, C—H⋯centroid = 128°], leading to the formation of columns parallel to the *b* axis. The close packing leads to the presence of a one-dimensional channel filled with partially occupied water mol­ecules engaged in O—H⋯O and O—H⋯I hydrogen bonds

## Related literature

For general background on the search for alternative treatments for microbial infections, see: Gomes *et al.* (2011 ▶); Alves *et al.* (2008 ▶); Carvalho *et al.* (2009 ▶). For the use of porphyrins as photosensitizers, see: Alves *et al.* (2009 ▶); Banfi *et al.* (2006 ▶); Merchat *et al.* (1996 ▶); Tomé *et al.* (2004 ▶); Yu *et al.* (2009 ▶). For general background on the work carried out by our group, see: Paz *et al.* (2002 ▶); Paz & Klinowski (2003 ▶); Shi *et al.* (2008 ▶).
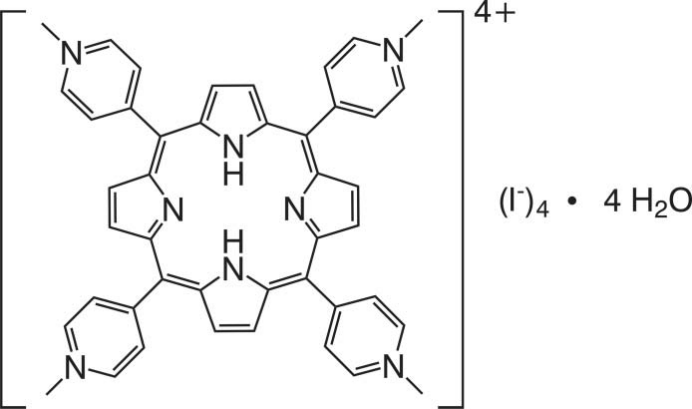


## Experimental

### 

#### Crystal data

C_44_H_38_N_8_^2+^·4I^−^·4H_2_O
*M* *_r_* = 1258.49
Monoclinic, 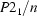

*a* = 24.3331 (4) Å
*b* = 6.5209 (1) Å
*c* = 30.5663 (5) Å
β = 95.025 (1)°
*V* = 4831.43 (13) Å^3^
*Z* = 4
Cu *K*α radiation
μ = 20.65 mm^−1^
*T* = 100 K
0.20 × 0.15 × 0.08 mm

#### Data collection

Bruker X8 KappaCCD APEXII diffractometer
Absorption correction: multi-scan (*SADABS*; Sheldrick, 1997 ▶) *T*_min_ = 0.104, *T*_max_ = 0.289
47661 measured reflections
7346 independent reflections
7013 reflections with *I* > 2σ(*I*)
*R*_int_ = 0.039

#### Refinement

*R*[*F* ^2^ > 2σ(*F* ^2^)] = 0.044
*wR*(*F* ^2^) = 0.110
*S* = 1.09
7346 reflections
563 parameters
6 restraints
H-atom parameters constrained
Δρ_max_ = 2.73 e Å^−3^
Δρ_min_ = −2.79 e Å^−3^

### 

Data collection: *APEX2* (Bruker, 2006 ▶); cell refinement: *APEX2*; data reduction: *SAINT-Plus* (Bruker, 2005 ▶); program(s) used to solve structure: *SHELXTL* (Sheldrick, 2008 ▶); program(s) used to refine structure: *SHELXTL*; molecular graphics: *DIAMOND* (Brandenburg, 2009 ▶); software used to prepare material for publication: *SHELXTL*.

## Supplementary Material

Crystal structure: contains datablock(s) global, I. DOI: 10.1107/S1600536811043844/tk5003sup1.cif

Structure factors: contains datablock(s) I. DOI: 10.1107/S1600536811043844/tk5003Isup2.hkl

Additional supplementary materials:  crystallographic information; 3D view; checkCIF report

## Figures and Tables

**Table 1:** Selected interatomic distances (Å)

O2*W*⋯O4*W*^i^	2.781 (15)
O2*W*⋯O6*W*^ii^	2.793 (15)
O3*W*⋯O5*W*^ii^	2.730 (19)
O3*W*⋯O6*W*	2.56 (2)
O4*W*⋯O5*W*	2.721 (19)
O5*W*⋯O6*W*^ii^	2.73 (2)
O1*W*⋯I2^iii^	3.565 (5)
O1*W*⋯I3^iii^	3.594 (5)
O2*W*⋯I3	3.684 (9)
O6*W*⋯I4^iv^	3.299 (18)
C13⋯I1	3.691 (6)
C14⋯I2^iv^	3.760 (6)
C44⋯I2	3.844 (7)
C22⋯I3^i^	3.882 (7)
C36⋯I4^v^	3.566 (5)

Symmetry codes: (i) 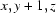; (ii) 
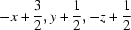
; (iii) 
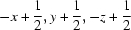
; (iv) 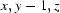; (v) 
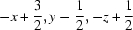
.

**Fig. 1.**
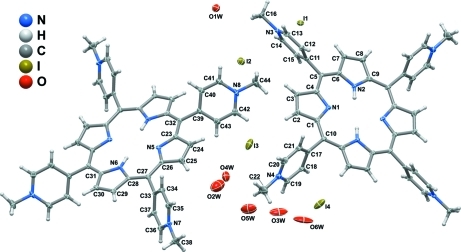
Molecular units comprising the asymmetric unit of the title compound. The porphyrin molecules have been completed (through inversion symmetry) for clarity. Displacement ellipsoids are drawn at the 50% probability level and the atomic labeling is provided for all non-hydrogen atoms comprising the asymmetric unit. Hydrogen atoms are represented as small spheres with arbitrary radius.

**Fig. 2.**
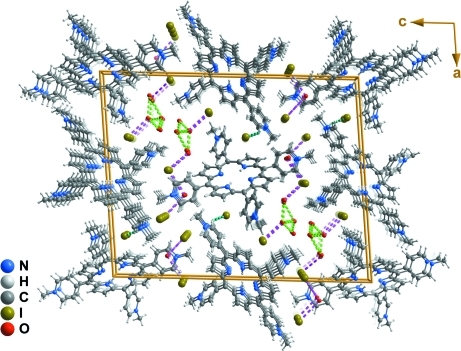
Crystal packing of the title compound viewed in perspective along the [010] direction of the unit cell. Hydrogen bonds involving only water molecules are depicted as dashed bright green lines and those involving water molecules and iodide anions are depicted as dashed pink lines. The C—H···I- weak hydrogen bond is depicted as a dashed green line.

**Fig. 3.**
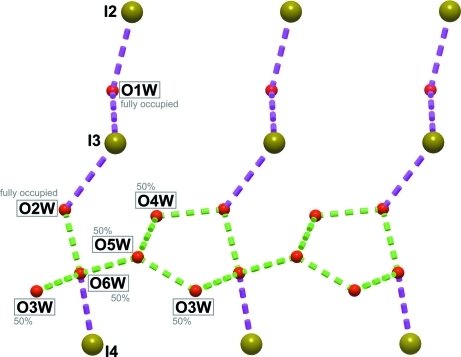
Portion of the hydrogen bonded zigzag chain involving iodide anions and water molecules of crystallization present in the crystal structure of the title compound. For clarity, the site occupancy of each water molecule is represented next to the label, and the symmetry codes used to generate equivalent atoms are omitted. For geometric details on the represented supramolecular interactions see Table 1.

## supplementary crystallographic information

### Comment

With the increasing number of antibiotic-resistant strains of microorganisms it is imperative to find alternative treatments for microbial infections (Alves *et al.*, 2008). Photodynamic therapy is a promising non-antibiotic approach to photoinactivate antibiotic-multi-resistant pathogenic microorganisms. The photodynamic destruction of microorganisms is based on the ability of certain photosensitizers, when activated by light, generate reactive oxygen species that are able to destroy or affect bacterial membranes (Carvalho *et al.*, 2009). For example, studies using cationic *meso*-substituted porphyrins acting as photosensitizers revealed interesting results in the destruction of *Gram*-positive and *Gram*-negative bacteria (Alves *et al.*, 2009; Banfi *et al.*, 2006; Merchat *et al.*, 1996). The compound 5,10,15,20-tetrakis(1-methylpyridinium-4-yl)porphyrin (Gomes *et al.*, 2011) is one of the most used photosensitizers in the photo-inactivation studies of microorganisms. Results have shown that the cationic conjugates are able to efficiently photosensitize different types of microorganisms (Gomes *et al.*, 2011; Tomé *et al.*, 2004; Alves *et al.*, 2008; Yu *et al.*, 2009). Following our on-going interest on organic crystals (Paz *et al.*, 2002; Paz & Klinowski, 2003) and in water clusters confined in organic/hybrid matrices (Shi *et al.*, 2008), here we wish to describe the crystal structure of the title compound.

The asymmetric unit (Fig. 1) of the title compound, (C_44_H_38_N_8_)I_4_^.^4H_2_O, comprises two distinct halves of centrosymmetric tetracationic porphyrin molecules, whose charge is balanced by four iodide anions, plus four water molecules of crystallization. The porphyrin rings are planar (with deviations from planarity smaller than *ca* 0.16 Å) subtending angles with the substituent pyridinium rings which range from *ca* 56 to *ca* 68°. The crystal structure is rich in weak supramolecular interactions such as π–π stacking and C—H···π interactions: there are two π–π stacking interactions between adjacent pyrrole rings [*Cg*···*Cg* distances of 3.762 (4) and 4.108 (4) Å], and there is a single C35—H35···π interaction [C···*Cg* of 3.522 (7) Å] between a hydrogen from the pyridinium ring and a pyrrole ring (interactions not shown). These supramolecular interactions contribute to the formation of columns of porphyrin molecules which are parallel to the *b* axis as depicted in Fig. 2. These columnar arrangements close pack in the *ac* plane leading to the formation of one-dimensional channels which, due to the need of close proximity of the iodide anions with the pyridinium rings, are instead filled with disordered water molecules of crystallization engaged in hydrogen bonding interactions (Table 1 and Figs 2 and 3). We note that a sole water molecule (O1W) is located between two iodide anions (I2 and I3) and outside the aforementioned channel, deeply embedded into the hydrophobic portion of the crystal structure. The remaining three water molecules are distributed among five independent crystallographic sites which may be involved in several O—H···O hydrogen bonding interactions as depicted in Fig. 3 (see Table 1 for geometric details). It is also interesting to note that only one iodide anion (I1) is not close to water molecules, participating instead in several short contacts with the porphyrin cations, among which a C—H···I^-^ weak hydrogen interaction (*d*_D···A_ = 3.691 (6) Å) arises as the strongest and more directional one (green dashed lines in Fig. 2). I2 to I4 are also involved in such type of interactions (not shown) as summarized in Table 1.

### Experimental

Crystals of the title compound have been isolated using the synthetic procedure described in detail by Gomes *et al.* (2011).

### Refinement

Hydrogen atoms bound to carbon and nitrogen were placed in idealized positions with C—H = 0.95 Å (aromatic) or 0.98 Å (terminal methyl groups), and N—H = 0.88 Å. These atoms were included in the final structural model in riding-motion approximation. The isotropic thermal displacement parameters for these atoms were fixed at 1.2 (for the aromatic H atoms) or 1.5 (for the terminal —CH_3_ moieties) times *U*_eq_ of the atom (C or N) to which they are attached.

Four water molecules of crystallization were found to be partially occupied and were included in the final structural model with fixed rates of occupancy of 50% (calculated from unrestrained refinement for the site occupancies). Hydrogen atoms associated with water molecules could not be located from difference Fourier maps and attempts to include these in calculated positions did not lead stable structural refinements. Nevertheless, the hydrogen atoms associated with these chemical entities have been included in the empirical formula of the title compound.

The structural model contains a large residual electron densities of 2.73 and -2.79 e^.^Å^-3^ located at 0.91 and 0.82 Å from the I4 atom, respectively. Attempts to include these peaks as a disordered iodide anion did not lead to sensible structural refinements.

### Crystal data

**Table:**

C_44_H_38_N_8_^2+^·4I^−^·4H_2_O	*F*(000) = 2440
*M**_r_* = 1258.49	*D*_x_ = 1.730 Mg m^−^^3^
Monoclinic, *P*2_1_/*n*	Cu *K*α radiation, λ = 1.54178 Å
Hall symbol: -P 2yn	Cell parameters from 8573 reflections
*a* = 24.3331 (4) Å	θ = 3.7–62.2°
*b* = 6.5209 (1) Å	µ = 20.65 mm^−^^1^
*c* = 30.5663 (5) Å	*T* = 100 K
β = 95.025 (1)°	Block, brown
*V* = 4831.43 (13) Å^3^	0.20 × 0.15 × 0.08 mm
*Z* = 4	

### Data collection

**Table:**

Bruker X8 KappaCCD APEXII diffractometer	7346 independent reflections
Radiation source: fine-focus sealed tube	7013 reflections with *I* > 2σ(*I*)
graphite	*R*_int_ = 0.039
ω and φ scans	θ_max_ = 62.7°, θ_min_ = 7.7°
Absorption correction: multi-scan (*SADABS*; Sheldrick, 1997)	*h* = −27→27
*T*_min_ = 0.104, *T*_max_ = 0.289	*k* = −7→7
47661 measured reflections	*l* = −22→34

### Refinement

**Table:**

Refinement on *F*^2^	Primary atom site location: structure-invariant direct methods
Least-squares matrix: full	Secondary atom site location: difference Fourier map
*R*[*F*^2^ > 2σ(*F*^2^)] = 0.044	Hydrogen site location: inferred from neighbouring sites
*wR*(*F*^2^) = 0.110	H-atom parameters constrained
*S* = 1.09	*w* = 1/[σ^2^(*F*_o_^2^) + (0.0481*P*)^2^ + 42.1978*P*] where *P* = (*F*_o_^2^ + 2*F*_c_^2^)/3
7346 reflections	(Δ/σ)_max_ = 0.001
563 parameters	Δρ_max_ = 2.73 e Å^−^^3^
6 restraints	Δρ_min_ = −2.79 e Å^−^^3^

### Special details

**Table:**

Geometry. All e.s.d.'s (except the e.s.d. in the dihedral angle between two l.s. planes) are estimated using the full covariance matrix. The cell e.s.d.'s are taken into account individually in the estimation of e.s.d.'s in distances, angles and torsion angles; correlations between e.s.d.'s in cell parameters are only used when they are defined by crystal symmetry. An approximate (isotropic) treatment of cell e.s.d.'s is used for estimating e.s.d.'s involving l.s. planes.
Refinement. Refinement of *F*^2^ against ALL reflections. The weighted *R*-factor *wR* and goodness of fit *S* are based on *F*^2^, conventional *R*-factors *R* are based on *F*, with *F* set to zero for negative *F*^2^. The threshold expression of *F*^2^ > σ(*F*^2^) is used only for calculating *R*-factors(gt) *etc*. and is not relevant to the choice of reflections for refinement. *R*-factors based on *F*^2^ are statistically about twice as large as those based on *F*, and *R*- factors based on ALL data will be even larger.

### Fractional atomic coordinates and isotropic or equivalent isotropic displacement parameters (Å^2^)

**Table:**

	*x*	*y*	*z*	*U*_iso_*/*U*_eq_	Occ. (<1)
N1	0.46552 (19)	0.1385 (8)	0.05010 (16)	0.0156 (11)	
N2	0.43689 (19)	−0.2216 (8)	−0.00352 (16)	0.0161 (11)	
H2	0.4640	−0.1320	−0.0009	0.019*	
N3	0.24687 (19)	−0.1678 (8)	0.12933 (15)	0.0145 (11)	
N4	0.6006 (2)	0.7920 (9)	0.17192 (18)	0.0247 (12)	
C1	0.4873 (2)	0.2957 (9)	0.0757 (2)	0.0160 (13)	
C2	0.4553 (2)	0.3298 (10)	0.1131 (2)	0.0178 (13)	
H2A	0.4630	0.4267	0.1360	0.021*	
C3	0.4128 (2)	0.1972 (10)	0.1089 (2)	0.0172 (13)	
H3	0.3839	0.1849	0.1278	0.021*	
C4	0.4196 (2)	0.0765 (9)	0.06957 (19)	0.0146 (12)	
C5	0.3859 (2)	−0.0908 (9)	0.05650 (19)	0.0137 (12)	
C6	0.3943 (2)	−0.2300 (9)	0.02283 (19)	0.0153 (13)	
C7	0.3613 (2)	−0.4026 (10)	0.0091 (2)	0.0173 (13)	
H7	0.3295	−0.4488	0.0222	0.021*	
C8	0.3828 (2)	−0.4894 (10)	−0.0258 (2)	0.0192 (14)	
H8	0.3687	−0.6060	−0.0417	0.023*	
C9	0.4311 (2)	−0.3731 (9)	−0.0346 (2)	0.0174 (13)	
C10	0.5360 (2)	0.4044 (9)	0.0686 (2)	0.0161 (13)	
C11	0.3363 (2)	−0.1240 (9)	0.08095 (19)	0.0133 (12)	
C12	0.2939 (2)	0.0224 (9)	0.07885 (19)	0.0139 (12)	
H12	0.2955	0.1386	0.0603	0.017*	
C13	0.2500 (2)	−0.0028 (9)	0.1039 (2)	0.0171 (13)	
H13	0.2217	0.0980	0.1030	0.021*	
C14	0.2850 (2)	−0.3158 (9)	0.13028 (19)	0.0155 (12)	
H14	0.2808	−0.4357	0.1473	0.019*	
C15	0.3306 (2)	−0.2950 (9)	0.10647 (19)	0.0165 (13)	
H15	0.3579	−0.3992	0.1078	0.020*	
C16	0.1992 (2)	−0.1914 (10)	0.1559 (2)	0.0211 (14)	
H16A	0.1680	−0.2503	0.1376	0.032*	
H16B	0.1888	−0.0569	0.1668	0.032*	
H16C	0.2093	−0.2825	0.1808	0.032*	
C17	0.5575 (2)	0.5474 (9)	0.1041 (2)	0.0155 (12)	
C18	0.6056 (2)	0.4934 (10)	0.1296 (2)	0.0201 (14)	
H18	0.6243	0.3703	0.1234	0.024*	
C19	0.6261 (3)	0.6164 (10)	0.1634 (2)	0.0229 (14)	
H19	0.6585	0.5771	0.1809	0.027*	
C20	0.5550 (3)	0.8516 (10)	0.1477 (2)	0.0249 (15)	
H20	0.5381	0.9781	0.1540	0.030*	
C21	0.5323 (3)	0.7323 (10)	0.1136 (2)	0.0212 (14)	
H21	0.4996	0.7753	0.0968	0.025*	
C22	0.6256 (3)	0.9247 (14)	0.2076 (3)	0.045 (2)	
H22A	0.6552	1.0066	0.1967	0.068*	
H22B	0.6407	0.8391	0.2322	0.068*	
H22C	0.5973	1.0162	0.2177	0.068*	
N5	0.51171 (18)	0.1474 (7)	0.44291 (15)	0.0119 (10)	
N6	0.54933 (18)	0.2175 (7)	0.53522 (15)	0.0120 (10)	
H6	0.5312	0.1189	0.5205	0.014*	
N7	0.6882 (2)	0.8716 (8)	0.43573 (17)	0.0179 (11)	
N8	0.4068 (2)	−0.1873 (8)	0.25635 (16)	0.0205 (12)	
C23	0.4912 (2)	0.0947 (9)	0.40114 (18)	0.0139 (12)	
C24	0.5102 (3)	0.2350 (10)	0.3689 (2)	0.0201 (14)	
H24	0.5004	0.2334	0.3381	0.024*	
C25	0.5444 (3)	0.3681 (9)	0.3913 (2)	0.0182 (13)	
H25	0.5647	0.4756	0.3793	0.022*	
C26	0.5441 (2)	0.3150 (9)	0.43721 (19)	0.0126 (12)	
C27	0.5742 (2)	0.4198 (9)	0.47193 (19)	0.0130 (12)	
C28	0.5731 (2)	0.3828 (9)	0.51656 (19)	0.0115 (12)	
C29	0.5950 (2)	0.5102 (9)	0.5519 (2)	0.0145 (13)	
H29	0.6135	0.6372	0.5490	0.017*	
C30	0.5850 (2)	0.4185 (9)	0.59024 (19)	0.0138 (12)	
H30	0.5943	0.4717	0.6189	0.017*	
C31	0.5579 (2)	0.2279 (9)	0.57998 (19)	0.0138 (12)	
C32	0.4584 (2)	−0.0811 (9)	0.39060 (19)	0.0131 (12)	
C33	0.6133 (2)	0.5840 (9)	0.45991 (18)	0.0131 (12)	
C34	0.5960 (2)	0.7561 (9)	0.4354 (2)	0.0170 (13)	
H34	0.5578	0.7760	0.4270	0.020*	
C35	0.6340 (3)	0.8974 (10)	0.4233 (2)	0.0191 (13)	
H35	0.6220	1.0132	0.4062	0.023*	
C36	0.7057 (2)	0.7111 (10)	0.4609 (2)	0.0198 (14)	
H36	0.7438	0.6983	0.4704	0.024*	
C37	0.6690 (2)	0.5647 (9)	0.4733 (2)	0.0160 (12)	
H37	0.6819	0.4514	0.4909	0.019*	
C38	0.7285 (3)	1.0258 (11)	0.4241 (2)	0.0287 (16)	
H38A	0.7390	1.1123	0.4496	0.043*	
H38B	0.7121	1.1112	0.4000	0.043*	
H38C	0.7613	0.9567	0.4148	0.043*	
C39	0.4407 (2)	−0.1190 (9)	0.34373 (19)	0.0141 (12)	
C40	0.3846 (2)	−0.1374 (9)	0.3289 (2)	0.0165 (13)	
H40	0.3575	−0.1257	0.3493	0.020*	
C41	0.3686 (3)	−0.1720 (9)	0.2855 (2)	0.0194 (13)	
H41	0.3306	−0.1852	0.2760	0.023*	
C42	0.4608 (3)	−0.1704 (10)	0.2694 (2)	0.0212 (14)	
H42	0.4871	−0.1805	0.2483	0.025*	
C43	0.4784 (3)	−0.1387 (9)	0.3127 (2)	0.0197 (14)	
H43	0.5168	−0.1302	0.3214	0.024*	
C44	0.3891 (3)	−0.2322 (11)	0.2096 (2)	0.0309 (17)	
H44A	0.4215	−0.2368	0.1927	0.046*	
H44B	0.3639	−0.1246	0.1978	0.046*	
H44C	0.3701	−0.3650	0.2075	0.046*	
I1	0.195450 (15)	0.46659 (6)	0.048331 (12)	0.01785 (12)	
I2	0.294854 (17)	0.24104 (7)	0.209298 (13)	0.02653 (13)	
I3	0.52507 (2)	0.35907 (8)	0.235129 (16)	0.04044 (15)	
I4	0.68215 (2)	0.99182 (7)	0.09052 (2)	0.04330 (16)	
O1W	0.09764 (19)	0.8556 (8)	0.20744 (16)	0.0320 (11)	
O2W	0.6112 (3)	0.6675 (15)	0.3155 (3)	0.088 (3)	
O3W	0.7269 (5)	0.367 (2)	0.2106 (6)	0.075 (4)	0.50
O4W	0.6235 (5)	0.0915 (17)	0.3155 (3)	0.040 (3)	0.50
O5W	0.7152 (6)	0.225 (2)	0.2792 (6)	0.074 (4)	0.50
O6W	0.7762 (5)	0.078 (2)	0.1729 (7)	0.093 (6)	0.50

### Atomic displacement parameters (Å^2^)

**Table:**

	*U*^11^	*U*^22^	*U*^33^	*U*^12^	*U*^13^	*U*^23^
N1	0.010 (2)	0.020 (3)	0.017 (3)	−0.001 (2)	0.0031 (19)	0.001 (2)
N2	0.010 (2)	0.021 (3)	0.018 (3)	−0.003 (2)	0.006 (2)	−0.002 (2)
N3	0.010 (2)	0.022 (3)	0.012 (2)	−0.001 (2)	0.0000 (19)	−0.002 (2)
N4	0.019 (3)	0.033 (3)	0.023 (3)	−0.008 (2)	0.004 (2)	−0.008 (3)
C1	0.012 (3)	0.018 (3)	0.018 (3)	0.004 (2)	0.005 (2)	−0.002 (3)
C2	0.019 (3)	0.018 (3)	0.017 (3)	0.001 (3)	0.007 (2)	−0.005 (3)
C3	0.013 (3)	0.021 (3)	0.018 (3)	0.000 (3)	0.006 (2)	0.001 (3)
C4	0.011 (3)	0.016 (3)	0.017 (3)	0.001 (2)	0.003 (2)	0.002 (3)
C5	0.010 (3)	0.016 (3)	0.015 (3)	0.004 (2)	0.005 (2)	0.004 (3)
C6	0.010 (3)	0.021 (3)	0.016 (3)	0.002 (2)	0.004 (2)	0.003 (3)
C7	0.010 (3)	0.023 (3)	0.019 (3)	−0.003 (3)	0.005 (2)	−0.002 (3)
C8	0.014 (3)	0.023 (3)	0.021 (3)	−0.004 (3)	0.002 (2)	−0.002 (3)
C9	0.014 (3)	0.019 (3)	0.019 (3)	0.000 (2)	−0.002 (2)	0.000 (3)
C10	0.009 (3)	0.018 (3)	0.022 (3)	0.001 (2)	0.004 (2)	0.003 (3)
C11	0.008 (3)	0.019 (3)	0.013 (3)	−0.004 (2)	0.001 (2)	−0.004 (2)
C12	0.012 (3)	0.015 (3)	0.014 (3)	0.000 (2)	0.003 (2)	0.004 (2)
C13	0.012 (3)	0.017 (3)	0.022 (3)	0.003 (2)	0.001 (2)	0.002 (3)
C14	0.017 (3)	0.017 (3)	0.012 (3)	−0.003 (3)	0.000 (2)	0.001 (2)
C15	0.012 (3)	0.019 (3)	0.018 (3)	0.003 (2)	−0.001 (2)	−0.002 (3)
C16	0.017 (3)	0.026 (4)	0.021 (3)	−0.004 (3)	0.008 (3)	0.002 (3)
C17	0.012 (3)	0.016 (3)	0.019 (3)	−0.003 (2)	0.006 (2)	0.000 (3)
C18	0.015 (3)	0.022 (3)	0.024 (3)	0.003 (3)	0.006 (3)	0.000 (3)
C19	0.019 (3)	0.029 (4)	0.020 (3)	0.002 (3)	−0.001 (3)	−0.006 (3)
C20	0.024 (3)	0.019 (3)	0.033 (4)	0.002 (3)	0.012 (3)	−0.006 (3)
C21	0.016 (3)	0.023 (3)	0.026 (4)	0.002 (3)	0.004 (3)	0.000 (3)
C22	0.040 (5)	0.048 (5)	0.046 (5)	−0.003 (4)	−0.001 (4)	−0.028 (4)
N5	0.010 (2)	0.012 (2)	0.014 (3)	−0.0003 (19)	0.0006 (19)	0.000 (2)
N6	0.010 (2)	0.015 (3)	0.010 (2)	−0.006 (2)	−0.0029 (18)	−0.001 (2)
N7	0.016 (3)	0.015 (3)	0.024 (3)	−0.006 (2)	0.007 (2)	−0.001 (2)
N8	0.031 (3)	0.017 (3)	0.011 (3)	−0.002 (2)	−0.007 (2)	−0.002 (2)
C23	0.014 (3)	0.015 (3)	0.012 (3)	0.000 (2)	0.000 (2)	0.000 (2)
C24	0.025 (3)	0.021 (3)	0.014 (3)	−0.004 (3)	0.000 (3)	0.001 (3)
C25	0.022 (3)	0.015 (3)	0.018 (3)	−0.005 (3)	0.000 (3)	0.002 (3)
C26	0.010 (3)	0.013 (3)	0.015 (3)	0.002 (2)	0.001 (2)	0.001 (2)
C27	0.012 (3)	0.010 (3)	0.017 (3)	−0.001 (2)	0.000 (2)	0.001 (2)
C28	0.006 (2)	0.012 (3)	0.016 (3)	0.000 (2)	−0.001 (2)	0.002 (2)
C29	0.010 (3)	0.015 (3)	0.019 (3)	0.000 (2)	0.000 (2)	−0.001 (2)
C30	0.014 (3)	0.013 (3)	0.014 (3)	0.000 (2)	−0.004 (2)	−0.001 (2)
C31	0.008 (3)	0.017 (3)	0.017 (3)	0.001 (2)	0.001 (2)	0.001 (2)
C32	0.013 (3)	0.014 (3)	0.012 (3)	0.002 (2)	0.001 (2)	−0.002 (2)
C33	0.012 (3)	0.014 (3)	0.013 (3)	−0.002 (2)	0.002 (2)	−0.005 (2)
C34	0.014 (3)	0.016 (3)	0.022 (3)	0.000 (2)	0.001 (2)	0.000 (3)
C35	0.021 (3)	0.014 (3)	0.023 (3)	0.001 (3)	0.003 (3)	0.000 (3)
C36	0.013 (3)	0.021 (3)	0.025 (3)	0.001 (3)	0.003 (3)	−0.003 (3)
C37	0.016 (3)	0.013 (3)	0.018 (3)	0.000 (2)	0.003 (2)	−0.003 (3)
C38	0.024 (4)	0.027 (4)	0.036 (4)	−0.009 (3)	0.009 (3)	0.002 (3)
C39	0.020 (3)	0.009 (3)	0.012 (3)	−0.001 (2)	−0.002 (2)	0.001 (2)
C40	0.017 (3)	0.014 (3)	0.017 (3)	−0.001 (2)	−0.005 (2)	0.005 (2)
C41	0.022 (3)	0.016 (3)	0.020 (3)	−0.001 (3)	−0.006 (3)	0.000 (3)
C42	0.030 (4)	0.018 (3)	0.016 (3)	−0.001 (3)	0.005 (3)	0.002 (3)
C43	0.020 (3)	0.017 (3)	0.021 (3)	−0.004 (3)	−0.003 (3)	0.000 (3)
C44	0.044 (4)	0.031 (4)	0.016 (3)	0.002 (3)	−0.006 (3)	−0.005 (3)
I1	0.0156 (2)	0.0166 (2)	0.0208 (2)	−0.00044 (15)	−0.00111 (15)	0.00148 (15)
I2	0.0291 (2)	0.0267 (2)	0.0232 (2)	−0.00046 (18)	−0.00126 (17)	0.00460 (17)
I3	0.0449 (3)	0.0435 (3)	0.0363 (3)	0.0162 (2)	0.0227 (2)	0.0111 (2)
I4	0.0381 (3)	0.0211 (2)	0.0761 (4)	0.0003 (2)	0.0356 (3)	0.0008 (2)
O1W	0.028 (3)	0.035 (3)	0.033 (3)	0.001 (2)	0.005 (2)	−0.006 (2)
O2W	0.060 (5)	0.111 (7)	0.094 (6)	0.011 (5)	0.015 (4)	0.032 (5)
O3W	0.026 (5)	0.054 (6)	0.141 (9)	0.004 (5)	−0.019 (6)	0.042 (7)
O4W	0.051 (6)	0.040 (6)	0.029 (6)	0.011 (5)	0.004 (5)	0.001 (5)
O5W	0.051 (8)	0.045 (8)	0.125 (13)	−0.007 (6)	0.004 (8)	−0.009 (8)
O6W	0.038 (7)	0.067 (10)	0.164 (17)	−0.012 (7)	−0.054 (9)	0.029 (10)

### Geometric parameters (Å, °)

**Table:**

N1—C1	1.368 (8)	N5—C26	1.367 (8)
N1—C4	1.373 (8)	N5—C23	1.373 (7)
N2—C6	1.367 (8)	N6—C31	1.367 (8)
N2—C9	1.370 (8)	N6—C28	1.371 (8)
N2—H2	0.8800	N6—H6	0.8800
N3—C13	1.334 (8)	N7—C36	1.347 (8)
N3—C14	1.337 (8)	N7—C35	1.350 (8)
N3—C16	1.480 (8)	N7—C38	1.471 (8)
N4—C20	1.337 (9)	N8—C42	1.344 (8)
N4—C19	1.339 (9)	N8—C41	1.347 (9)
N4—C22	1.481 (9)	N8—C44	1.484 (8)
C1—C10	1.414 (8)	C23—C32	1.417 (8)
C1—C2	1.454 (8)	C23—C24	1.450 (9)
C2—C3	1.347 (9)	C24—C25	1.348 (9)
C2—H2A	0.9500	C24—H24	0.9500
C3—C4	1.458 (9)	C25—C26	1.446 (9)
C3—H3	0.9500	C25—H25	0.9500
C4—C5	1.401 (9)	C26—C27	1.413 (8)
C5—C6	1.401 (9)	C27—C28	1.388 (8)
C5—C11	1.490 (8)	C27—C33	1.499 (8)
C6—C7	1.426 (9)	C28—C29	1.428 (8)
C7—C8	1.353 (9)	C29—C30	1.358 (9)
C7—H7	0.9500	C29—H29	0.9500
C8—C9	1.442 (9)	C30—C31	1.429 (8)
C8—H8	0.9500	C30—H30	0.9500
C9—C10^i^	1.384 (9)	C31—C32^ii^	1.394 (9)
C10—C9^i^	1.384 (9)	C32—C31^ii^	1.394 (9)
C10—C17	1.489 (9)	C32—C39	1.480 (8)
C11—C15	1.375 (9)	C33—C37	1.386 (8)
C11—C12	1.402 (8)	C33—C34	1.394 (9)
C12—C13	1.378 (9)	C34—C35	1.379 (9)
C12—H12	0.9500	C34—H34	0.9500
C13—H13	0.9500	C35—H35	0.9500
C14—C15	1.385 (9)	C36—C37	1.382 (9)
C14—H14	0.9500	C36—H36	0.9500
C15—H15	0.9500	C37—H37	0.9500
C16—H16A	0.9800	C38—H38A	0.9800
C16—H16B	0.9800	C38—H38B	0.9800
C16—H16C	0.9800	C38—H38C	0.9800
C17—C18	1.393 (9)	C39—C43	1.383 (9)
C17—C21	1.395 (9)	C39—C40	1.404 (8)
C18—C19	1.367 (9)	C40—C41	1.368 (9)
C18—H18	0.9500	C40—H40	0.9500
C19—H19	0.9500	C41—H41	0.9500
C20—C21	1.376 (10)	C42—C43	1.372 (9)
C20—H20	0.9500	C42—H42	0.9500
C21—H21	0.9500	C43—H43	0.9500
C22—H22A	0.9800	C44—H44A	0.9800
C22—H22B	0.9800	C44—H44B	0.9800
C22—H22C	0.9800	C44—H44C	0.9800
			
O2W···O4W^iii^	2.781 (15)	O2W···I3	3.684 (9)
O2W···O6W^iv^	2.793 (15)	O6W···I4^vi^	3.299 (18)
O3W···O5W^iv^	2.730 (19)	C13···I1	3.691 (6)
O3W···O6W	2.56 (2)	C14···I2^vi^	3.760 (6)
O4W···O5W	2.721 (19)	C44···I2	3.844 (7)
O5W···O6W^iv^	2.73 (2)	C22···I3^iii^	3.882 (7)
O1W···I2^v^	3.565 (5)	C36···I4^vii^	3.566 (5)
O1W···I3^v^	3.594 (5)		
			
C1—N1—C4	105.2 (5)	C26—N5—C23	104.4 (5)
C6—N2—C9	110.0 (5)	C31—N6—C28	110.2 (5)
C6—N2—H2	125.0	C31—N6—H6	124.9
C9—N2—H2	125.0	C28—N6—H6	124.9
C13—N3—C14	121.3 (5)	C36—N7—C35	120.6 (5)
C13—N3—C16	119.4 (5)	C36—N7—C38	119.1 (5)
C14—N3—C16	119.2 (5)	C35—N7—C38	120.2 (5)
C20—N4—C19	121.1 (6)	C42—N8—C41	120.8 (5)
C20—N4—C22	120.4 (6)	C42—N8—C44	119.7 (6)
C19—N4—C22	118.5 (6)	C41—N8—C44	119.5 (5)
N1—C1—C10	125.1 (5)	N5—C23—C32	124.4 (5)
N1—C1—C2	111.0 (5)	N5—C23—C24	111.2 (5)
C10—C1—C2	123.8 (6)	C32—C23—C24	124.2 (5)
C3—C2—C1	106.6 (5)	C25—C24—C23	106.3 (5)
C3—C2—H2A	126.7	C25—C24—H24	126.8
C1—C2—H2A	126.7	C23—C24—H24	126.8
C2—C3—C4	106.5 (5)	C24—C25—C26	106.4 (5)
C2—C3—H3	126.7	C24—C25—H25	126.8
C4—C3—H3	126.7	C26—C25—H25	126.8
N1—C4—C5	125.8 (5)	N5—C26—C27	124.0 (5)
N1—C4—C3	110.7 (5)	N5—C26—C25	111.5 (5)
C5—C4—C3	123.3 (5)	C27—C26—C25	124.5 (5)
C6—C5—C4	126.4 (5)	C28—C27—C26	126.9 (5)
C6—C5—C11	116.9 (5)	C28—C27—C33	115.7 (5)
C4—C5—C11	116.7 (5)	C26—C27—C33	117.4 (5)
N2—C6—C5	125.1 (5)	N6—C28—C27	126.2 (5)
N2—C6—C7	107.1 (5)	N6—C28—C29	106.6 (5)
C5—C6—C7	127.8 (5)	C27—C28—C29	127.1 (5)
C8—C7—C6	108.4 (5)	C30—C29—C28	108.2 (5)
C8—C7—H7	125.8	C30—C29—H29	125.9
C6—C7—H7	125.8	C28—C29—H29	125.9
C7—C8—C9	107.7 (6)	C29—C30—C31	108.0 (5)
C7—C8—H8	126.1	C29—C30—H30	126.0
C9—C8—H8	126.1	C31—C30—H30	126.0
N2—C9—C10^i^	126.6 (6)	N6—C31—C32^ii^	125.8 (5)
N2—C9—C8	106.7 (5)	N6—C31—C30	106.8 (5)
C10^i^—C9—C8	126.7 (6)	C32^ii^—C31—C30	127.4 (5)
C9^i^—C10—C1	126.2 (6)	C31^ii^—C32—C23	126.5 (5)
C9^i^—C10—C17	116.7 (5)	C31^ii^—C32—C39	115.8 (5)
C1—C10—C17	116.7 (5)	C23—C32—C39	117.7 (5)
C15—C11—C12	117.9 (5)	C37—C33—C34	118.4 (5)
C15—C11—C5	122.0 (5)	C37—C33—C27	119.1 (5)
C12—C11—C5	120.1 (5)	C34—C33—C27	122.5 (5)
C13—C12—C11	119.8 (5)	C35—C34—C33	120.2 (5)
C13—C12—H12	120.1	C35—C34—H34	119.9
C11—C12—H12	120.1	C33—C34—H34	119.9
N3—C13—C12	120.4 (5)	N7—C35—C34	120.2 (6)
N3—C13—H13	119.8	N7—C35—H35	119.9
C12—C13—H13	119.8	C34—C35—H35	119.9
N3—C14—C15	120.2 (6)	N7—C36—C37	121.0 (5)
N3—C14—H14	119.9	N7—C36—H36	119.5
C15—C14—H14	119.9	C37—C36—H36	119.5
C11—C15—C14	120.2 (6)	C36—C37—C33	119.6 (6)
C11—C15—H15	119.9	C36—C37—H37	120.2
C14—C15—H15	119.9	C33—C37—H37	120.2
N3—C16—H16A	109.5	N7—C38—H38A	109.5
N3—C16—H16B	109.5	N7—C38—H38B	109.5
H16A—C16—H16B	109.5	H38A—C38—H38B	109.5
N3—C16—H16C	109.5	N7—C38—H38C	109.5
H16A—C16—H16C	109.5	H38A—C38—H38C	109.5
H16B—C16—H16C	109.5	H38B—C38—H38C	109.5
C18—C17—C21	117.7 (6)	C43—C39—C40	117.1 (5)
C18—C17—C10	118.3 (5)	C43—C39—C32	121.7 (5)
C21—C17—C10	124.0 (5)	C40—C39—C32	121.2 (5)
C19—C18—C17	120.4 (6)	C41—C40—C39	120.8 (6)
C19—C18—H18	119.8	C41—C40—H40	119.6
C17—C18—H18	119.8	C39—C40—H40	119.6
N4—C19—C18	120.4 (6)	N8—C41—C40	120.0 (6)
N4—C19—H19	119.8	N8—C41—H41	120.0
C18—C19—H19	119.8	C40—C41—H41	120.0
N4—C20—C21	121.0 (6)	N8—C42—C43	120.9 (6)
N4—C20—H20	119.5	N8—C42—H42	119.6
C21—C20—H20	119.5	C43—C42—H42	119.6
C20—C21—C17	119.4 (6)	C42—C43—C39	120.4 (6)
C20—C21—H21	120.3	C42—C43—H43	119.8
C17—C21—H21	120.3	C39—C43—H43	119.8
N4—C22—H22A	109.5	N8—C44—H44A	109.5
N4—C22—H22B	109.5	N8—C44—H44B	109.5
H22A—C22—H22B	109.5	H44A—C44—H44B	109.5
N4—C22—H22C	109.5	N8—C44—H44C	109.5
H22A—C22—H22C	109.5	H44A—C44—H44C	109.5
H22B—C22—H22C	109.5	H44B—C44—H44C	109.5
			
C4—N1—C1—C10	178.6 (6)	C26—N5—C23—C32	175.1 (5)
C4—N1—C1—C2	1.9 (6)	C26—N5—C23—C24	−1.3 (6)
N1—C1—C2—C3	−2.4 (7)	N5—C23—C24—C25	2.6 (7)
C10—C1—C2—C3	−179.2 (6)	C32—C23—C24—C25	−173.8 (6)
C1—C2—C3—C4	1.9 (7)	C23—C24—C25—C26	−2.7 (7)
C1—N1—C4—C5	−175.4 (6)	C23—N5—C26—C27	−179.2 (5)
C1—N1—C4—C3	−0.6 (6)	C23—N5—C26—C25	−0.5 (6)
C2—C3—C4—N1	−0.9 (7)	C24—C25—C26—N5	2.1 (7)
C2—C3—C4—C5	174.0 (6)	C24—C25—C26—C27	−179.2 (6)
N1—C4—C5—C6	3.3 (10)	N5—C26—C27—C28	−5.3 (9)
C3—C4—C5—C6	−170.8 (6)	C25—C26—C27—C28	176.2 (6)
N1—C4—C5—C11	−176.9 (5)	N5—C26—C27—C33	172.4 (5)
C3—C4—C5—C11	9.0 (8)	C25—C26—C27—C33	−6.1 (8)
C9—N2—C6—C5	−176.4 (6)	C31—N6—C28—C27	177.9 (5)
C9—N2—C6—C7	3.0 (7)	C31—N6—C28—C29	−3.5 (6)
C4—C5—C6—N2	−1.5 (10)	C26—C27—C28—N6	11.1 (9)
C11—C5—C6—N2	178.8 (5)	C33—C27—C28—N6	−166.7 (5)
C4—C5—C6—C7	179.3 (6)	C26—C27—C28—C29	−167.3 (6)
C11—C5—C6—C7	−0.5 (9)	C33—C27—C28—C29	14.9 (8)
N2—C6—C7—C8	−2.3 (7)	N6—C28—C29—C30	1.0 (6)
C5—C6—C7—C8	177.1 (6)	C27—C28—C29—C30	179.6 (5)
C6—C7—C8—C9	0.7 (7)	C28—C29—C30—C31	1.7 (6)
C6—N2—C9—C10^i^	175.5 (6)	C28—N6—C31—C32^ii^	−177.0 (5)
C6—N2—C9—C8	−2.5 (7)	C28—N6—C31—C30	4.5 (6)
C7—C8—C9—N2	1.1 (7)	C29—C30—C31—N6	−3.8 (6)
C7—C8—C9—C10^i^	−177.0 (6)	C29—C30—C31—C32^ii^	177.7 (6)
N1—C1—C10—C9^i^	1.2 (10)	N5—C23—C32—C31^ii^	−0.5 (9)
C2—C1—C10—C9^i^	177.5 (6)	C24—C23—C32—C31^ii^	175.4 (6)
N1—C1—C10—C17	−170.6 (6)	N5—C23—C32—C39	−178.4 (5)
C2—C1—C10—C17	5.7 (9)	C24—C23—C32—C39	−2.5 (9)
C6—C5—C11—C15	65.1 (7)	C28—C27—C33—C37	58.3 (7)
C4—C5—C11—C15	−114.7 (6)	C26—C27—C33—C37	−119.7 (6)
C6—C5—C11—C12	−115.8 (6)	C28—C27—C33—C34	−122.1 (6)
C4—C5—C11—C12	64.4 (7)	C26—C27—C33—C34	59.9 (8)
C15—C11—C12—C13	3.5 (8)	C37—C33—C34—C35	2.9 (9)
C5—C11—C12—C13	−175.7 (5)	C27—C33—C34—C35	−176.7 (6)
C14—N3—C13—C12	−2.2 (8)	C36—N7—C35—C34	−1.7 (9)
C16—N3—C13—C12	−180.0 (5)	C38—N7—C35—C34	−178.0 (6)
C11—C12—C13—N3	−1.4 (9)	C33—C34—C35—N7	−1.0 (9)
C13—N3—C14—C15	3.7 (8)	C35—N7—C36—C37	2.5 (9)
C16—N3—C14—C15	−178.6 (5)	C38—N7—C36—C37	178.9 (6)
C12—C11—C15—C14	−2.1 (8)	N7—C36—C37—C33	−0.5 (9)
C5—C11—C15—C14	177.1 (5)	C34—C33—C37—C36	−2.1 (9)
N3—C14—C15—C11	−1.4 (9)	C27—C33—C37—C36	177.5 (5)
C9^i^—C10—C17—C18	−64.2 (8)	C31^ii^—C32—C39—C43	−120.9 (6)
C1—C10—C17—C18	108.5 (6)	C23—C32—C39—C43	57.2 (8)
C9^i^—C10—C17—C21	116.3 (7)	C31^ii^—C32—C39—C40	58.8 (7)
C1—C10—C17—C21	−71.0 (8)	C23—C32—C39—C40	−123.0 (6)
C21—C17—C18—C19	1.6 (9)	C43—C39—C40—C41	−0.4 (9)
C10—C17—C18—C19	−177.9 (6)	C32—C39—C40—C41	179.8 (6)
C20—N4—C19—C18	−0.3 (10)	C42—N8—C41—C40	0.5 (9)
C22—N4—C19—C18	−177.4 (7)	C44—N8—C41—C40	177.8 (6)
C17—C18—C19—N4	−1.1 (10)	C39—C40—C41—N8	−0.5 (9)
C19—N4—C20—C21	1.2 (10)	C41—N8—C42—C43	0.5 (9)
C22—N4—C20—C21	178.2 (7)	C44—N8—C42—C43	−176.9 (6)
N4—C20—C21—C17	−0.6 (10)	N8—C42—C43—C39	−1.4 (9)
C18—C17—C21—C20	−0.7 (9)	C40—C39—C43—C42	1.3 (9)
C10—C17—C21—C20	178.8 (6)	C32—C39—C43—C42	−178.9 (6)

Symmetry codes: (i) −*x*+1, −*y*, −*z*; (ii) −*x*+1, −*y*, −*z*+1; (iii) *x*, *y*+1, *z*; (iv) −*x*+3/2, *y*+1/2, −*z*+1/2;  (v) −*x*+1/2, *y*+1/2, −*z*+1/2; (vi) *x*, *y*−1, *z*; (vii) −*x*+3/2, *y*−1/2, −*z*+1/2.
